# Experimental investigation of the sequence injection effect of sea water and smart water into an offshore carbonate reservoir for enhanced oil recovery

**DOI:** 10.1038/s41598-024-55440-8

**Published:** 2024-02-26

**Authors:** Amir Hossein Saeedi Dehaghani, Reza Daneshfar

**Affiliations:** 1 Department of Petroleum Engineering, Faculty of Chemical Engineering, Tarbiat Modares University, Tehran, Iran; 2 Department of Petroleum Engineering, Ahwaz Faculty of Petroleum Engineering, Petroleum University of Technology (PUT), Ahwaz, Iran

**Keywords:** Enhanced oil recovery, Smart water, Wettability, Micromodel, Sequence effect, Crude oil, Hydrology

## Abstract

This study explores enhanced oil recovery (EOR) strategies, with a focus on carbonate reservoirs constituting over 60% of global oil discoveries. While “smart water” injection proves effective in EOR for carbonate reservoirs, offshore application challenges arise due to impractical volumes for injection. To address this, we propose a novel continuous injection approach, systematically investigating it on a laboratory scale using the Iranian offshore reservoir, Sivand. Thirty-six contact angle tests and twelve flooding experiments are meticulously conducted, with key ions, potassium, and sulfate, playing pivotal roles. Optimal wettability alteration is observed at 4 times potassium ion concentration in 0–2 times sulfate concentrations, driven by ionic strength and charge interactions. Conversely, at 3–5 times sulfate concentrations, the optimal contact angle shifts to 2 times potassium ion concentration, suggesting a mechanism change linked to increasing sulfate ion ionicity. A significant wettability alteration, evidenced by a 132.8° decrease, occurs in seawater with a twofold concentration of potassium ions and a fivefold concentration of sulfate ions. Micromodel experiments introduce an innovative alternation of smart water and seawater injections. The first scenario, smart water followed by seawater injection, reveals negligible post-seawater injection oil recovery changes. In contrast, the second scenario yields a maximum recovery of 7.9%. The first scenario, however, boasts superior overall sweep efficacy, reaching approximately 43%. This research expands understanding of smart water and seawater injection in EOR, presenting a viable solution for optimizing offshore carbonate reservoir recovery. The insights contribute to evolving EOR methodologies, emphasizing tailored strategies for varying reservoir conditions.

## Introduction

In recent years, the escalating demand for energy has prompted researchers and oil companies to venture into the exploration of new oil fields, a venture fraught with substantial expenses^1–3^. Simultaneously, the issue of stability has taken center stage in industrial regions across the global community^4–6^. It is imperative to chart a course that maximizes efficiency while minimizing costs and environmental impact^7,8^.

Smart water technologies have emerged as pivotal tools in optimizing oil production processes. These technologies involve the use of advanced sensors and data analytics to monitor and manage reservoir conditions effectively^9^. Furthermore, it’s noteworthy that over half of the world’s oil reservoirs, including the majority in Iran, belong to the carbonate type, and they are currently at their midpoint in terms of lifespan^10,11^. As these reservoirs undergo production, a decline in pressure ensues, ultimately leading to a reduction in production output^12–14^.

The implementation of smart water injection techniques has shown promising results in enhancing oil recovery from carbonate reservoirs. By manipulating the chemical composition of injected water, researchers aim to mitigate reservoir pressure decline and prolong the productive life of these reservoirs^15^. In this pursuit, aligning our efforts with sustainable practices is crucial, considering the environmental impact of traditional oil extraction methods^16^. Balancing the increasing energy demand with eco-friendly solutions remains a critical challenge for the future. Oil recovery from these reservoirs poses significant challenges, attributed to factors such as low permeability, mixed-to-oil-wetness of the reservoir rock, a high density of natural fractures, and a notable degree of heterogeneity^17–19^. To overcome these challenges, advanced smart water flooding techniques have emerged as a viable solution in the oil industry. This involves the injection of specifically tailored water with enhanced properties to maximize oil recovery^20^.

In addressing the complexities of oil extraction from such reservoirs, the implementation of pressure maintenance and enhanced oil recovery methods becomes imperative^21–23^. Smart water flooding stands out as a cost-effective method compared to alternative techniques for increasing oil recovery. The economic viability of this approach has garnered significant attention from researchers, contributing to its widespread adoption^24^. Scientific studies have shown that the use of smart water, enriched with certain ions and surfactants, can alter the reservoir conditions favorably, reducing oil-wetness and enhancing fluid flow through the rock pores^25,26^. By tailoring the composition of injected smart water, researchers aim to address the specific challenges associated with each reservoir, optimizing the recovery process.

In recent years, the industry has witnessed the successful application of smart water flooding after the initial seawater flooding phase, significantly boosting oil production rates^27–31^. This underscores the method’s effectiveness in extracting additional reserves while minimizing costs. The continued exploration and refinement of smart water technologies hold promise for further advancements in the field of enhanced oil recovery^32–34^. In recent years, extensive research has been conducted on the subject of wettability alteration, utilizing ion concentrations to enhance oil recovery^35–39^. Smart water, a key player in this context, disrupts the equilibrium of the primary crude oil, salt, and rock system by precisely adjusting the composition of injected liquid ions^40,41^.

Scientific investigations indicate that smart water induces wettability alteration, influencing capillary pressure and relative permeability in porous media, thereby stimulating oil recovery^42^. Moreover, the modulation of the salinity in the injected fluid is widely recognized for its substantial impact on wettability, directly influencing the efficiency of oil recovery^43,44^. Smart water flooding primarily leverages wetting alteration as the most crucial mechanism influencing oil production^45,46^. Recent discussions have explored alternative mechanisms, but none have been universally accepted as the principal mechanism. The fundamental objective of employing smart water flooding is to strategically modify and optimize the composition of combined ions, thereby altering the wettability properties of the reservoir^47–49^. This cost-effective and environmentally friendly method stands out for its avoidance of expensive chemicals that may otherwise harm the reservoir^50–52^. It is noteworthy that numerous studies have explored the interaction of smart water with various chemicals, including surfactants^53–57^, CO_2_^58,59^, polymer^60,61^, and nanoparticles^62–64^, within carbonate rocks. However, the primary focus of this paper centers on investigations utilizing smart water alone to enhance oil recovery from carbonate rocks.

In recent years, many researchers have delved into the use of smart water as a fluid to boost oil recovery in carbonate reservoirs. A pivotal study by Strand et al. conducted spontaneous imbibition tests on chalky samples, revealing the crucial role of temperature in facilitating the diffusion of effective ions into porous mediums and, consequently, enhancing oil extraction^65^. Further exploration into this area could provide valuable insights into optimizing smart water formulations for specific reservoir conditions. In a related study, Zhang et al. identified the significance of Ca^2+^ and SO_4_^2−^ ions in the spontaneous uptake of smart water at elevated temperatures^66^. These findings, corroborated by Fattahi et al., underscore the importance of understanding the role of individual ions in the smart water composition for effective oil recovery^67^. Their study highlighted that a reduction in NaCl salt concentration in smart water could lead to a 5% decrease in oil recovery. Analyzing the symbiotic interaction of active ions in smart water, Sung et al. explored the impact of Na^+^ and SO_4_^2−^ ions on altering the wettability of oil-wet limestone plug samples during spontaneous imbibition experiments^68^. Their results revealed a direct correlation between decreased Na^+^ concentration and increased SO_4_^2−^ concentration with enhanced oil recovery. In experiments conducted by Alameri and colleagues, seawater flooding on carbonate plug samples led to a reduction in Ca^2+^, Mg^2+^, Cl^−^, and SO_4_^2−^ ions at the outlet. Simultaneously, the wettability of samples transformed from oil-wet to intermediate-wet^69^. This study underscores the dynamic changes in reservoir conditions induced by smart water flooding. Shirazi et al. further contributed to the understanding of spontaneous imbibition by concluding that SO_4_^2−^ plays a more significant role in wettability alteration than Ca^2+^ in carbonate rock samples^70^. Additionally, they noted a minor role for Mg^2+^ in this process. Collectively, these studies highlight the intricate interplay of ions within smart water, offering valuable insights for optimizing strategies to enhance oil recovery in carbonate reservoirs.

Despite numerous studies by various researchers aimed at enhancing oil recovery from carbonate rocks using smart water, there has been a notable gap in research concerning the sequence of smart water injection in relation to seawater. This becomes particularly crucial in offshore reservoirs where access to vast volumes of seawater is readily available, making it a potential game-changer. Recognizing this, our paper takes a pioneering step by putting this idea into practice at the laboratory scale.

The primary objective of this study is to assess the impact of the injection sequence of smart water and seawater on recovered oil. In subsequent sections, we aim to identify the most effective injection sequence and the optimal injection composition. This will be achieved through carefully designed experiments that involve measuring contact angles and assessing flooding dynamics, taking into account the influence of specific ions present in smart water. The experimental design will include variations in the injection sequence and composition, allowing us to systematically evaluate the outcomes and identify the conditions that lead to the most efficient oil recovery. The measurement of contact angles will provide insights into wettability alterations induced by different injection sequences and compositions, while flooding experiments will offer a quantitative assessment of oil displacement efficiency. In conclusion, this research aims to bridge the existing gap in understanding the impact of smart water and seawater injection sequences on oil recovery from carbonate rocks. The insights gained from this study could pave the way for more informed and effective strategies in offshore oil reservoir management.

## Materials and methods

Various salts, obtained from Merck Company, were employed to prepare formation water, seawater, and smart water with varying concentrations. In this study, Persian Gulf water served as seawater, while the formation water used in the experiments was extracted from the Sivand formation. The characteristics and ion compositions of these two water samples are detailed in Table 1.

**Table 1:** The concentration of ions in seawater and formation water.

Brine	SO_4_^2−^	Cl^−^	Mg^2+^	Ca^2+^	Na^+^	HCO_3_^−^	Sr^+^	K^+^	Total salinity (mg/L)
Sea water concentration (mg/L)	3110	21,410	1632	440	12,000	166	3	399	39,160
Formation water concentration (mg/L)	635	73,942	759	5032	42,215	579	547	1986	125,695

The research utilized oil with a density of 830 kg/m^3^ and a viscosity of 0.00497 Pa.s. The components of this oil are outlined in Table 2. Additionally, carbonate rock sourced from the Sivand offshore formation in the Siri oil district of Iran was employed in the experiments. The composition of this rock, as determined by the XRF test, is presented in Table 3.

**Table 2:** Crude oil components.

N_2_	CO_2_	C_1_	C_2_	C_3_	iC_4_	nC_4_	iC_5_	nC_5_	C_6_	C_7_	C_8_	C_9_^+^
Negligible	Negligible	0.01	0.02	0.01	0.78	1.83	3.99	5.95	6.82	6.22	8.21	66.15

**Table 3:** The results of the XRF test.

Material	CaO	Na_2_O	MgO	Al_2_O_3_	SiO_2_	P_2_O_5_	SO_3_	Cl	K_2_O	MnO	Fe_2_O_3_	Sr	L.O.I
Percent	55.024	0.041	0.363	0.076	0.224	0.008	0.110	0.017	0.015	0.072	0.914	0.047	43.09

**Figure 1:**
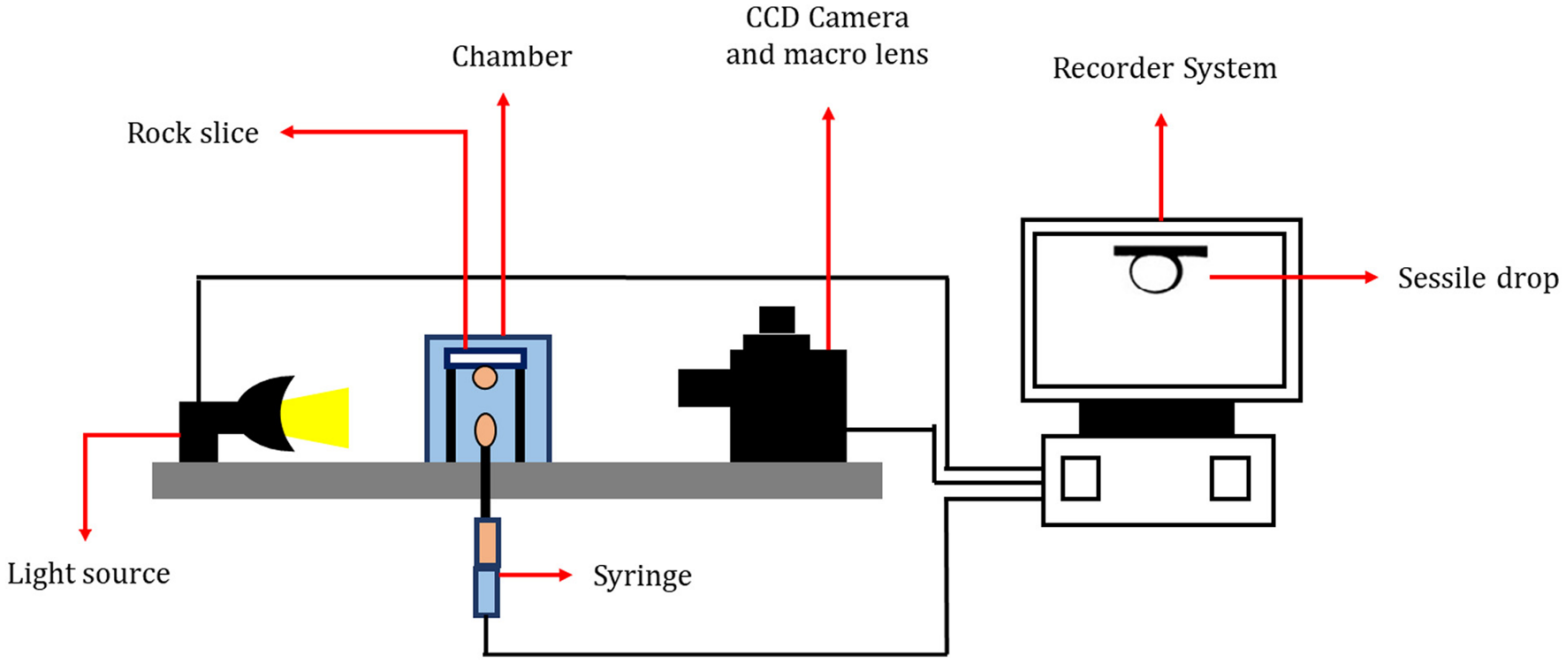
A schematic of the experimental setup of the contact angle measurement.

The determination of contact angles between the rock surface and oil involved a comprehensive experimental procedure utilizing a sessile drop contact angle measuring device, as outlined in Fig. 1. The initial step included a meticulous cleaning process for the rock slices, employing toluene and methanol to eliminate excess fat and solid particles within the rock matrix. To assess the initial wettability, these cleaned rock slices underwent a 1-week immersion in synthetic formation water at ambient temperature. Subsequently, to restore the original wettability, the slices underwent a 2-week aging process in crude oil at 90 °C^71,72^. By conducting the test at this stage, it has been ensured that all samples have become completely oil-wet. The final wettability was achieved by placing the slices in various aqueous solutions. Notably, after the preparation of smart water solutions, the chips derived from the reservoir rock were immersed in these solutions for contact angle tests over a period of 5 days.

The experimental setup incorporated a macro lens, a dedicated light source, and a rock and fluid chamber. Controlled delivery of the aqueous solution was ensured through an injection syringe, and visual data capture was accomplished using a Charge-coupled device (CCD) camera. The entire process was monitored and analyzed using specialized software, providing a thorough exploration of contact angles under diverse conditions.

To ensure meticulous micromodel measurements, a comprehensive set of experimental procedures was implemented, prioritizing simplicity and clarity. To counteract gravitational effects on fluid flow, the micromodel was intentionally positioned horizontally^73^, maintaining a steady injection rate of approximately 0.01 ml/h. Employing a microfluidic device, we fashioned a two-dimensional porous medium with intricately etched narrow conduits (pores) on its surface, mirroring the pore structure of carbonate rock. Two holes were drilled at opposite corners of the glass plane to serve as input and output conduits for precise fluid injection and production, a departure from traditional micromodels. Our design facilitated the examination of sweeping performance, emulating a five-spot injection pattern commonly adopted in classical Enhanced Oil Recovery studies^74,75^.

**Figure 2:**
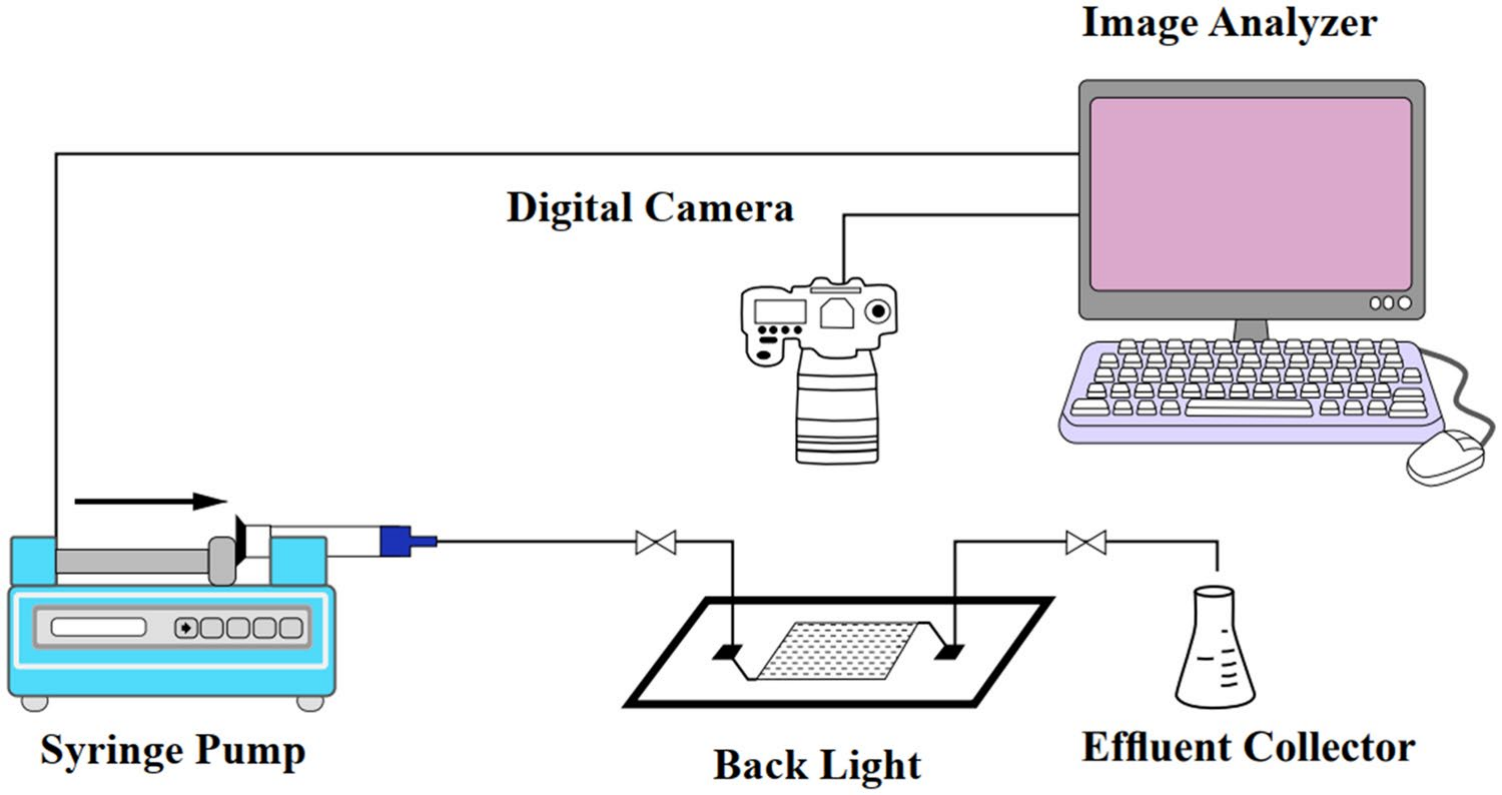
A schematic of a micro-model injection setup.

**Figure 3:**
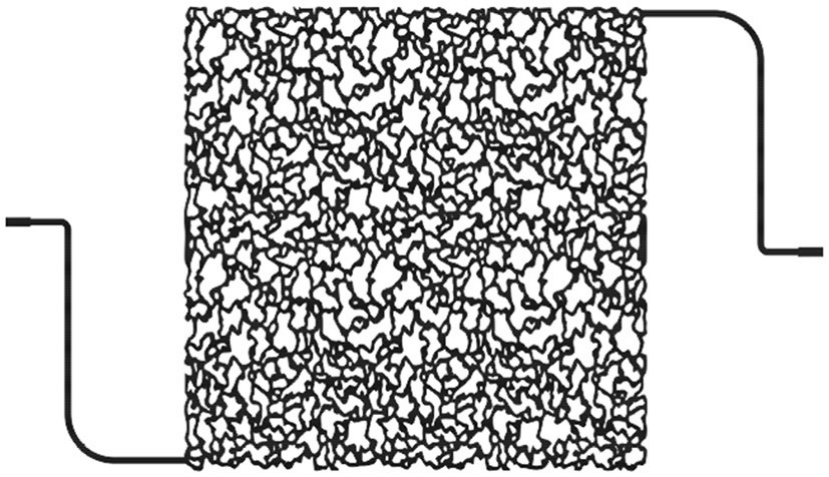
Micromodel design used in experiments.

The micromodel injection setup, illustrated in Fig. 2, incorporated essential components like a syringe pump, computer, fluid collectors, a camera, and an injection syringe. Creating the micromodel involved preliminary steps of glass preparation and laser treatment for fluid passage design. Subsequently, a glass of identical size was placed on it and fused together in a furnace under specific temperature steps, ranging from 300 to 700 °C and back to 100 °C within a 5-h timeframe. The resulting model, featuring a porosity of 37% and a pore volume (PV) of 0.22 mL, was visually presented in Fig. 3. Prior to usage, the micromodel underwent a crucial oil-wetting process using a solution comprising 95% toluene and 5% hexamethyldisilane, ensuring a strong oil-wet state^76^. These streamlined yet comprehensive steps underscored the reliability and relevance of the micromodel measurements in this research. Experiments were conducted in the micromodel under atmospheric conditions at a temperature of 25 °C to determine displacement characteristics.

## Results and discussion

In this section, we analyze the results of the experiments. Initially, we scrutinize the outcomes of the contact angle experiments, followed by an examination of the results derived from the micromodel experiments. The micromodel experiments were conducted with the objective of elucidating oil recovery mechanisms.

### Analysis of contact angle tests

As delineated in the preceding section, focusing on the washing and aging of chip samples, the requisite procedure for conducting these tests involves immersing the samples in smart water solutions for a duration of 5 days. Smart water solutions are meticulously formulated by manipulating the concentrations of sulfate and potassium ions in seawater. The foundational water for these experiments emanates from the Persian Gulf. The determined concentrations for sulfate and potassium ions are itemized in Table 4. These concentrations are presented as multiples in seawater and are slated for utilization in subsequent sections of the study.

**Table 4:** Different concentrations of potassium and sulfate ions in seawater.

Multiplied concentration (times)	0	0.25	0.5	1	2	3	4	5
Potassium concentration (mg/L)	0	100	200	399	798	–	1596	–
Sulfate concentration (mg/L)	0	–	–	3110	6220	12,440	24,880	49,760

Table 5 presents data illustrating contact angle experiments conducted at six distinct concentrations of sulfate and potassium in seawater. This experimental design resulted in a total of 36 tests. It is crucial to emphasize that three independent tests were executed at each concentration, and the averages of these trials are presented in the aforementioned Table.

**Table 5:** The results of contact angle tests.

Multiply the concentration of these two ions		Potassium					
		0 times	0.25 times	0.5 times	1 times	2 times	4 times
Sulfate	0 times	77.4	74.2	73.4	71.4	73	69.2
	1 times	74.2	70.2	69.2	70	68.2	66
	2 times	68.3	67.2	65.1	64.5	65.4	61.6
	3 times	64	61.6	63.9	61.2	58.1	63.5
	4 times	63.7	60.9	56.9	56.7	54.1	54.2
	5 times	56.6	56.6	52.1	49.9	47.2	48.7

**Figure 4:**
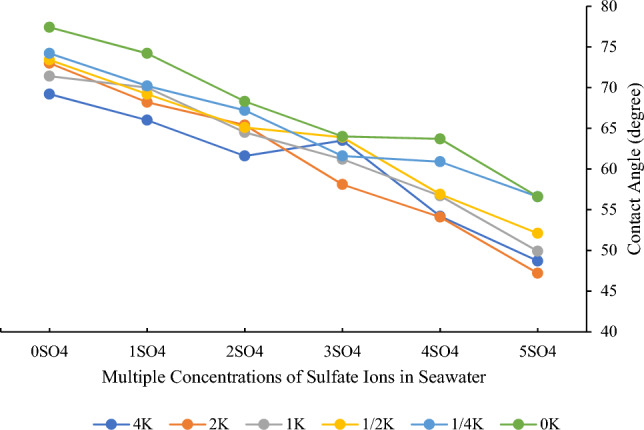
Changes in contact angle at different concentrations of sulfate ions in seawater and constant concentrations of potassium ions.

**Figure 5:**
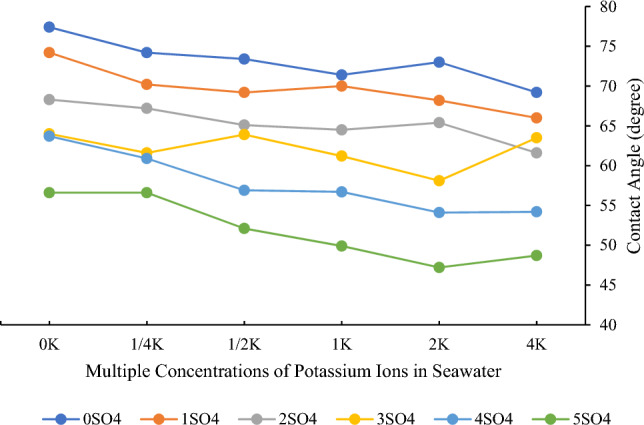
Changes in contact angle at different concentrations of potassium ions in seawater and constant concentrations of sulfate ions.

Two distinct sets of graphs illustrate the data. In Fig. 4, the concentration of potassium ions remains constant while the concentration of sulfate ions is varied, enabling an exploration of the effects of sulfate ions. In Fig. 5, in contrast to the preceding analysis, the focus shifts to the impact of altering the concentration of potassium ions while maintaining a constant sulfate ion concentration. These graphical representations serve as visual evidence of the distinct effects of sulfate and potassium ions on contact angle variations in smart water formulations.

Figure 4 illustrates the impact of increasing sulfate ion concentration in seawater. As depicted in the diagram, augmenting the sulfate ion concentration leads to a reduction in the contact angle, inducing a shift in wettability towards a more hydrophilic state. Notably, the observed outcomes with sulfate ions in these carbonate rocks align with findings from experiments conducted on chalky rocks in recent years^77,78^.

The reduction in contact angle is attributed to a fundamental mechanism involving the absorption of negatively charged fatty acids from crude oil onto the positively charged surface of the carbonate rock. This process is intricately linked to the concentration of SO_4_^2−^ ions in the solution, which diminishes the positive charge on the rock’s surface.

**Figure 6:**
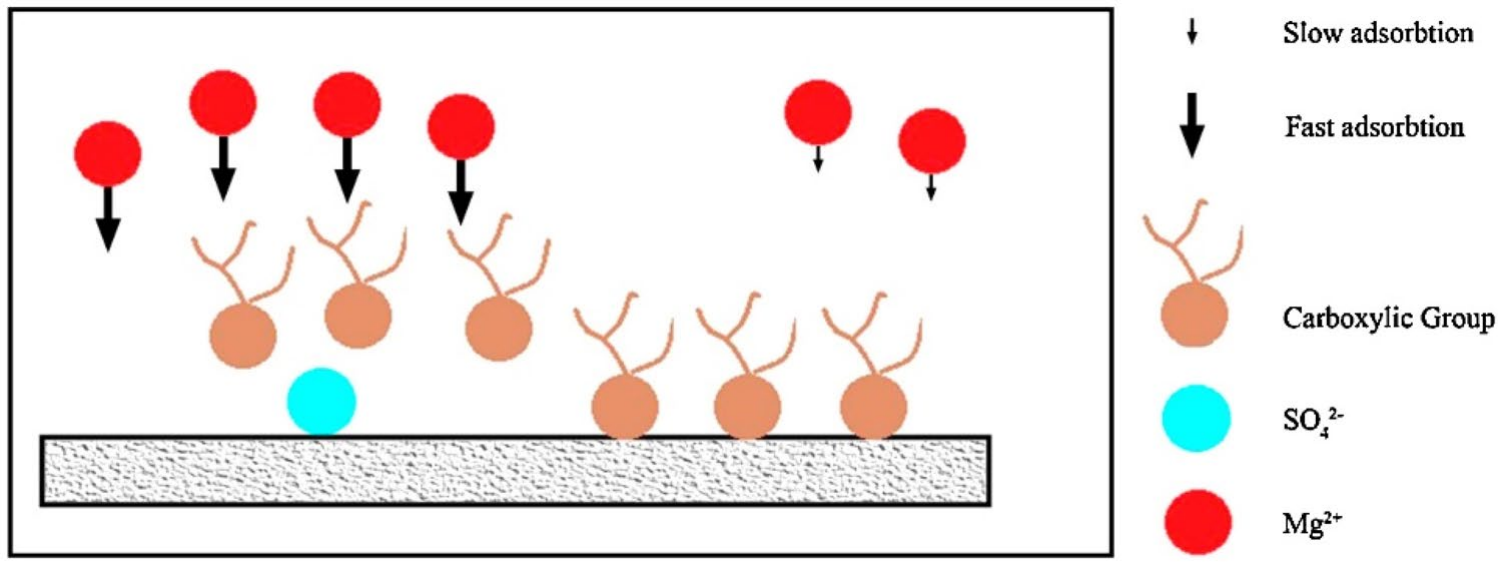
The role of sulfate ions in changing wettability with smart water on the surface of carbonate rocks.

Figure 6 provides a schematic representation of this mechanism, elucidating the role of sulfate ions in reducing electrostatic repulsion force and enhancing the activity of magnesium and calcium in proximity to the rock’s surface. The presence of sodium ions in seawater induces minor changes in the contact angle, suggesting the potential for more favorable results by eliminating sodium ions^79^.

Upon careful observation and analysis of Fig. 5, it becomes evident that potassium ions do not exert a pronounced influence as determining ions. The contact angle exhibits minimal variation with changes in potassium ion concentration. While this ion does contribute to a moderate reduction in the contact angle, inducing a shift toward hydrophilicity, the magnitude of these alterations is insubstantial. Consequently, it is not advisable to implement changes in potassium ion concentration on a field scale due to the negligible impact on wettability.

The limited impact of potassium ions is associated with the Debye length, a factor in lyophobic colloidal systems. The potassium ion, with lower ionic strength compared to sodium, results in a thicker double electric layer, facilitating easier separation of oil from the surface and inducing a shift towards hydrophilicity^80^.

To elucidate the mechanism underlying this phenomenon, it is imperative to provide an introductory context. In lyophobic colloidal systems, where two components coexist, such as oil components dispersing in water, their interaction is contingent upon the thickness of the dispersed layer, commonly referred to as the Debye length. Notably, the Debye length is inversely proportional to the ionic strength^81^.

The potassium ion, constituting the potassium chloride salt, possesses a lower ionic strength compared to other cations, such as sodium. This discrepancy results in a longer Debye length, thereby generating a thicker double electric layer. This increased thickness facilitates the easier separation of oil from the surface, consequently inducing a shift in wettability towards a more hydrophilic state.

The intriguing variation in optimal potassium ion concentration based on sulfate ion levels in smart water formulations can be attributed to the intricate interplay of these ions and their distinct effects on the wettability of carbonate rock. In the concentrations of 0, 1, and 2 times the sulfate ion, where the best wettability alteration was achieved at 4 times the potassium ion concentration, the mechanisms at play are associated with the ionic strength and charge interactions on the mineral surface^82^. At higher potassium ion concentrations, the increased ionic strength can facilitate the screening of repulsive forces, leading to a more favorable adsorption of ions on the carbonate rock surface^83,84^. This, in turn, can induce a shift towards a water-wet state by promoting the separation of fatty acids from the surface and altering the rock’s charge characteristics.

Conversely, in the concentrations of 3, 4, and 5 times the sulfate ion, where the optimal contact angle was observed at 2 times the potassium ion concentration, the change in mechanism is likely linked to the increasing ionicity of sulfate ions. Higher sulfate concentrations may enhance their adsorption on the mineral surface, resulting in alterations in surface charge and, subsequently, wettability^85^. The specific strength of sulfate ions in modifying the surface properties of carbonate rock at these levels might necessitate a different balance with potassium ions, leading to the observed shift in optimal conditions. The interplay between sulfate and potassium ions, their varying impacts on ionic strength and surface charge, and their ability to influence the adsorption and desorption of surfactants collectively contribute to the nuanced results observed in the contact angle tests.

In conclusion, the dynamic interaction of sulfate and potassium ions with carbonate rock surfaces, modulating ionic strength, surface charge, and surfactant behavior, underpins the observed variations in optimal potassium ion concentration. This understanding is crucial for tailoring smart water formulations in enhanced oil recovery processes, providing insights into the complexities of wettability modification in response to different ion concentrations.

### Analysis of micromodel tests

In this section, micromodel experiments are conducted, employing a dual design approach. In the first group, smart water—determined to yield optimal results in contact angle experiments—is injected into the micromodel. Subsequently, after breakthrough, seawater is introduced, and the ensuing increase in recovery is meticulously examined. In the second case, in stark contrast to the preceding scenario, the experimental sequence involves the initial injection of seawater. Following breakthrough, the subsequent injection comprises smart water, and the subsequent recovery is quantitatively assessed.

As evident from the contact angle data, the wettability exhibited negligible alteration in response to variations in potassium ion concentration. Conversely, noteworthy changes in the contact angle were observed with fluctuations in sulfate ion concentration. Subsequently, in this section, we identify the optimal solutions for conducting micromodel experiments. In the concentrations of 0, 1, and 2 times the sulfate ion, the optimal contact angle was achieved with 4 times the potassium ion concentration, while in the concentrations of 3, 4, and 5 times the sulfate ion, the optimal contact angle was obtained with 2 times the potassium ion concentration. These six concentrations are subsequently recommended for utilization in the micromodel experiments.

In each experimental iteration, the initial step involves injecting the formation water into the micromodel. Following this, the injection of oil continues until the water saturation attains the desired residual level. Subsequently, injections of smart water and seawater are conducted in a predefined sequence. Smart water comprises six distinct solutions, each characterized by the concentrations specified in the preceding paragraph. The fluid injection rates in both approaches were consistent, remaining at 0.01 mL/h. Following the completion of the first stage, the second stage commences promptly. In both scenarios, the injection of the second fluid persists until there is no further oil flowing from the outlet side of the micromodel.

In every experimental trial, a photograph is captured prior to introducing the smart water solution into the oil-saturated micromodel. Subsequently, another image is taken at the culmination of the brine injection process, precisely at the moment of breakthrough. Following this procedural step, the two images undergo scrutiny using MATLAB software. Through analysis of the black and white segments within the photos, the quantity of extracted oil is computed. The oil recovery is then determined by dividing this extracted volume by the initial volume of oil.

#### Implementing the first scenario and examining its performance

In this section, experiments are conducted involving the sequential injection of smart water followed by seawater into the micromodel. Analysis of the recovery results presented in Table 6 reveals a discernible trend: an increase in sulfate ion concentration correlates with an elevation in oil recovery. This augmentation in recovery manifests even during the initial stage of injection when only smart water is introduced, suggesting a plausible alteration in wettability induced by sulfate ions. Furthermore, it is essential to emphasize that sulfate ions play a crucial role in modifying the chemical interactions at the oil-water-rock interface, leading to improved oil recovery. The interaction involves sulfate ions reacting with fatty acids on the porous media’s wall, displacing them and effecting a shift in wettability, which enhances the oil recovery process^86^.

**Table 6:** Increased recovery in the first mode injection (first smart water then seawater).

Smart water solution	Sw4K0SO_4_	Sw4K1SO_4_	Sw4K2SO_4_	Sw2K3SO_4_	Sw2K4SO_4_	Sw2K5SO_4_
Efficiency of the first stage of injection (percent)	22.1	32.2	35.4	37	41.3	43.4
Efficiency of the second stage of injection (percent)	23	32.9	36.5	38.4	42.7	43.7
Increase the efficiency of the second stage compared to the first stage (percent)	0.9	0.7	1.1	1.4	1.4	0.3

Another noteworthy observation is the reduction in the growth rate of recovery when potassium ion concentration decreases from four times to two times. Although this reduction is not profound, it indicates that diminishing potassium ion concentration modestly reduces recovery, possibly influenced by the relatively low ionic strength of potassium ions. This reduction in recovery could be attributed to the diminished effectiveness of potassium ions in altering the wettability when present in lower concentrations. The alteration in ionic strength may influence the electrostatic forces at the oil-water-rock interface, impacting the recovery efficiency^87^. Upon completing the initial smart water injection phase, subsequent seawater injection yields no significant increase in recovery. The observed increase in recovery of the second stage compared to the first stage falls within the range of 0.7–1.4 percent.

**Figure 7:**
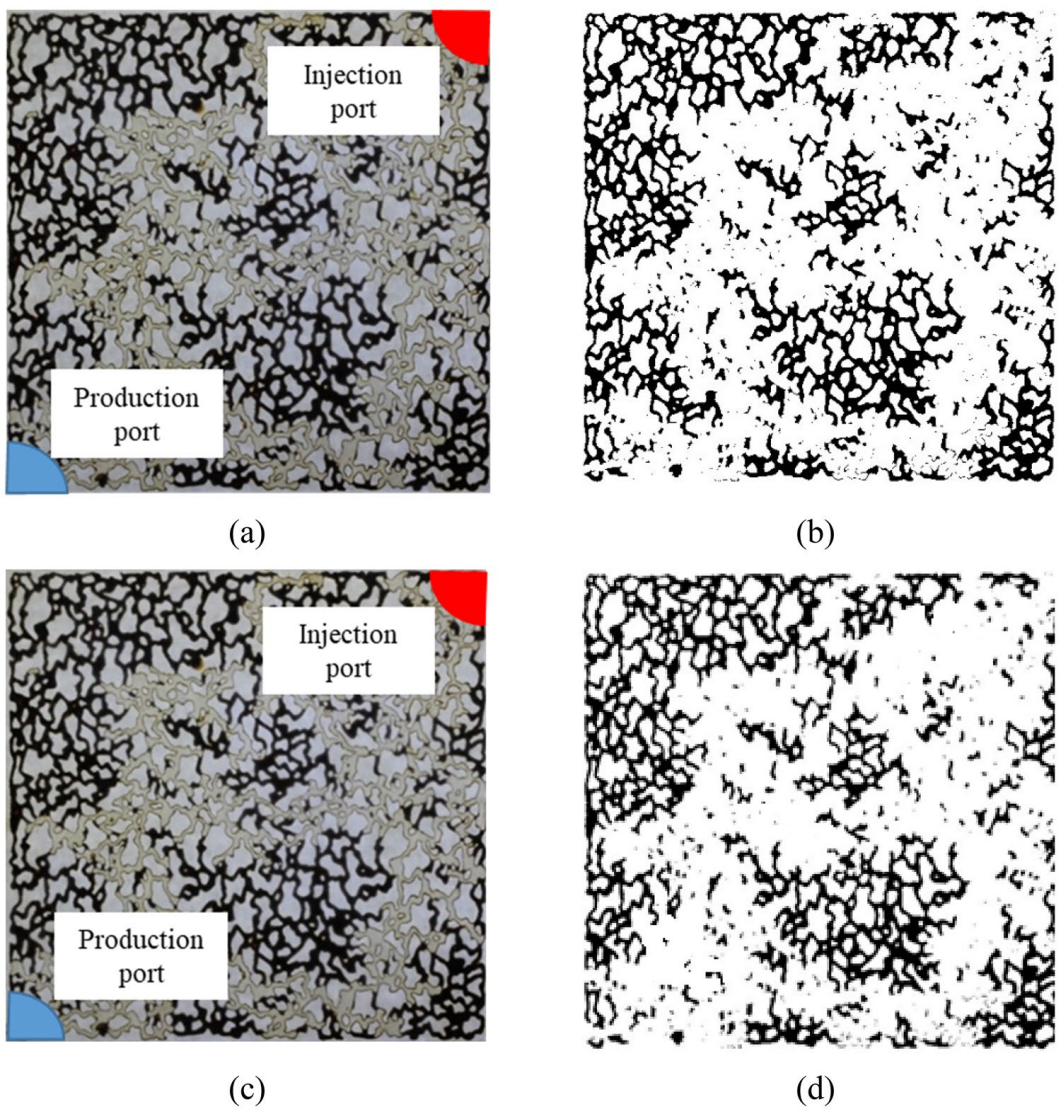
First mode injection (smart water and seawater injection in the first and second stages, respectively): (**a**) The image taken at the moment of breakthrough; (**b**) Analysis of image (**a**) in MATLAB; (**c**) The image taken at the end of the experiment; (**d**) Analysis of image (**c**) in MATLAB.

Based on the results obtained from this injection phase, it becomes evident that seawater follows a path previously traversed by smart water, exerting minimal influence on wettability. Consequently, the observed increase in recovery attributable to seawater injection is inconsequential. This further supports the conclusion that the sulfate ions in the smart water play a pivotal role in modifying the wettability, and subsequent seawater injection does not significantly contribute to this modification. Data analysis reveals that the second injection stage is suboptimal, with the initial smart water injection stage proving to be sufficient for achieving the desired outcomes. Figure 7 illustrates the initial injection phase, wherein the micromodel is subjected to smart water and seawater in the primary and secondary injection stages, respectively. The injection and production ports are shown using red and blue colors, respectively. The smart water solution has sulfate and potassium ion concentrations four times and two times higher than seawater, respectively. Upon MATLAB analysis of the figure, the oil recovery after the initial stage is determined to be 41.3%. Subsequently, Fig. 7a visually depicts the oil displacement by the smart water solution, reaching breakthrough moment. Figure 7b represents the processed image in black and white using MATLAB software. Figure 7c showcases the final image of the second injection stage in the micromodel, involving seawater injection in the continuation of the experiment. Notably, this stage exhibits a 1.4% increase in recovery, reaching a total of 42.7%.

#### Implementing the second scenario and examining its performance

Now, let’s consider the second injection mode in the micromodel. In this phase, seawater is initially injected, followed by the injection of smart water after breakthrough. The outcomes indicate that seawater contributes to a modest increase in recovery; however, this augmentation is not deemed significant. It’s noteworthy that seawater alone does not exhibit a substantial impact on wettability, supporting the hypothesis that the observed recovery increase is primarily driven by the chemical interactions induced by the sulfate ions in the smart water^88^. The limited recovery and unsatisfactory sweep coefficient observed during seawater flooding (prior to smart water injection) can be attributed to the inherent challenges associated with seawater’s effectiveness in modifying wettability. Seawater, lacking the elevated sulfate ion concentration found in smart water, may exhibit limited capability to induce substantial alterations in the oil-water-rock interface^89^. The lower ionic strength of seawater and its potential inadequacy to overcome existing interfacial forces contribute to reduced wettability modification, resulting in suboptimal recovery during this phase of the injection process^90^. The subsequent injection of smart water, rich in sulfate ions, becomes crucial for achieving significant recovery enhancements by addressing these limitations in wettability modification.

Subsequent to achieving breakthrough, the injection of smart water, whose optimal ion concentrations have been previously determined, results in a notable enhancement in recovery. The detailed results of the second injection mode are presented in Table 7, where the first stage involves seawater injection, and the second stage involves smart water injection.

**Table 7:** Increased recovery in the second mode injection (first seawater then smart water).

Smart water solution	Sw4K0SO_4_	Sw4K1SO_4_	Sw4K2SO_4_	Sw2K3SO_4_	Sw2K4SO_4_	Sw2K5SO_4_
Efficiency of the first stage of injection (percent)	18.7	17	18.1	17.9	17.2	17.5
Efficiency of the second stage of injection (percent)	19.8	21.2	23.8	24.2	25.1	24.3
Increase the efficiency of the second stage compared to the first stage (percent)	1.1	4.2	5.7	6.2	7.9	6.8

An important observation made during the experiments was the absence of a consistent upward trend in recovery with increasing solution concentrations. This lack of a linear relationship between solution concentrations and recovery could be attributed to the complex interplay of various factors, including the intricate chemistry of the oil-water-rock system and the influence of solution composition on wettability^91^. The underlying cause for this phenomenon was identified in the oil-wetting stage of the micromodel. During this phase, a slightly prolonged exposure of the micromodel to a solution comprising toluene and hexamethyldisilane results in a heightened state of oil-wetness, consequently leading to a reduction in recovery. This intricacy should be duly acknowledged and considered in the interpretation of experimental outcomes.

**Figure 8:**
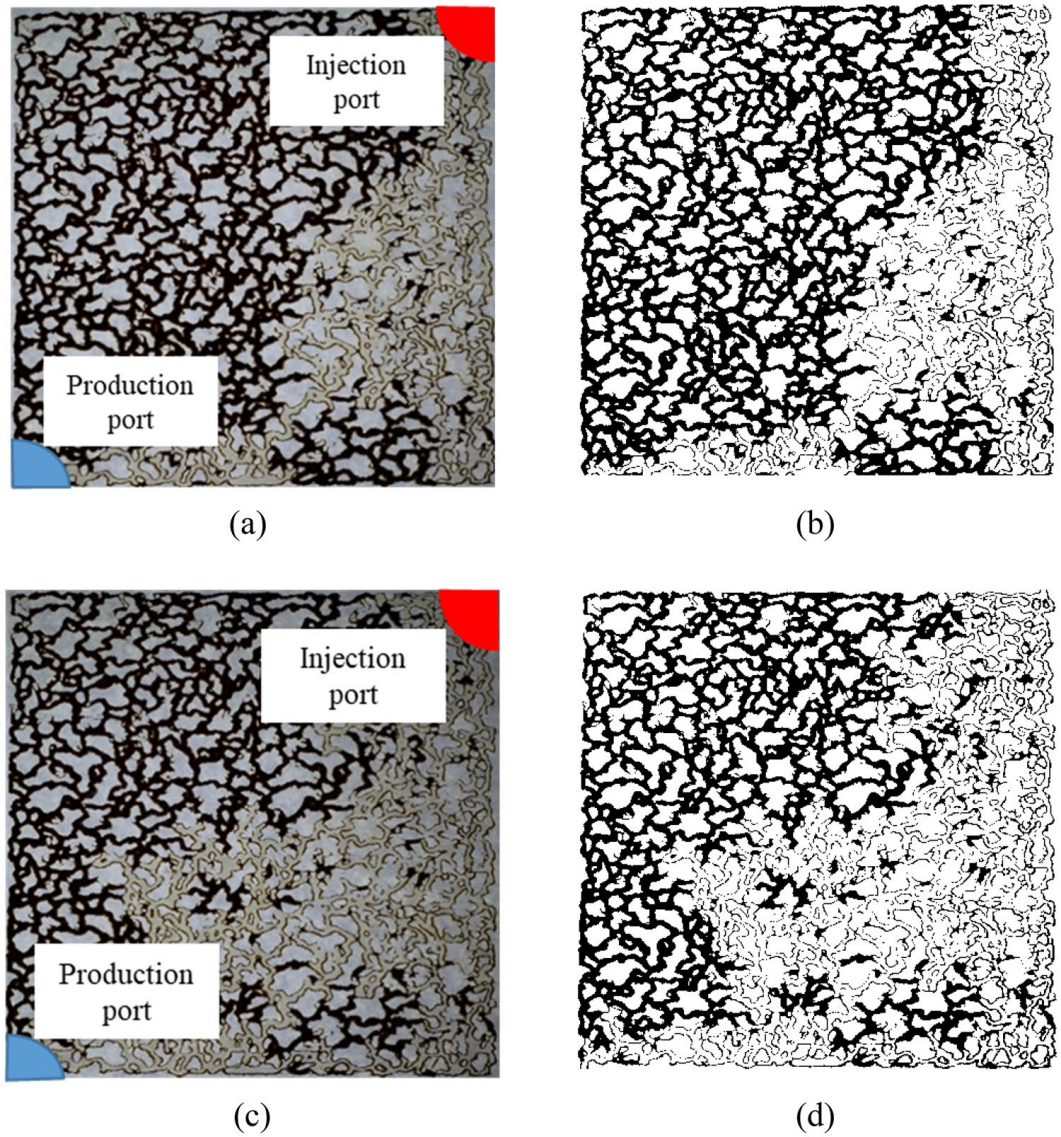
Second mode injection (seawater and smart water injection in the first and second stages, respectively): (**a**) The image taken at the moment of breakthrough; (**b**) Analysis of image (**a**) in MATLAB; (**c**) The image taken at the end of the experiment; (**d**) Analysis of image (**c**) in MATLAB.

Furthermore, the impact of solution concentration on recovery is influenced by the intricate interfacial phenomena occurring at the microscopic level, including the adsorption of ions onto mineral surfaces and changes in interfacial tension^92,93^. These factors collectively contribute to the observed variations in recovery efficiency. Following the injection of seawater and attaining the breakthrough point, smart water is introduced into the micromodel. During these stages, a noteworthy increase in oil recovery, ranging from approximately 1.1–7.9 percent, has been observed. In the instance of seawater devoid of sulfate ions, a marginal increase in recovery, around 1%, is noted. This modest enhancement is attributed to the presence of potassium ions, which, despite their relatively limited impact, exhibit some effectiveness in the absence of sulfate ions. The absence of sulfate ions results in a diminished reaction with fatty acids on the porous media’s wall, underscoring the significance of sulfate ions in the observed recovery increase. The augmentation in recovery is directly proportional to the sulfate ion concentration in the solution, with the most substantial increases observed for states featuring 4 and 5 times sulfate ion injections, yielding a 7.9% and 6.8% recovery increase, respectively. This heightened effect is anticipated, particularly in the case of the solution with fourfold sulfate ions and twofold potassium ions, as this solution demonstrated exemplary outcomes in the contact angle tests. In this context, it significantly altered the wettability of the micromodel, thereby contributing to a substantial enhancement in recovery. Figure 8a,b depict an image capturing the moment of seawater injection at the breakthrough time. Subsequently, Figure 8c,d showcase the micromodel image following the completion of the injection of a smart water solution featuring fourfold sulfate ions and twofold potassium ions. Notably, this injection occurred subsequent to the initial seawater injection phase.

### Microscopic analysis of micromodels

**Figure 9:**
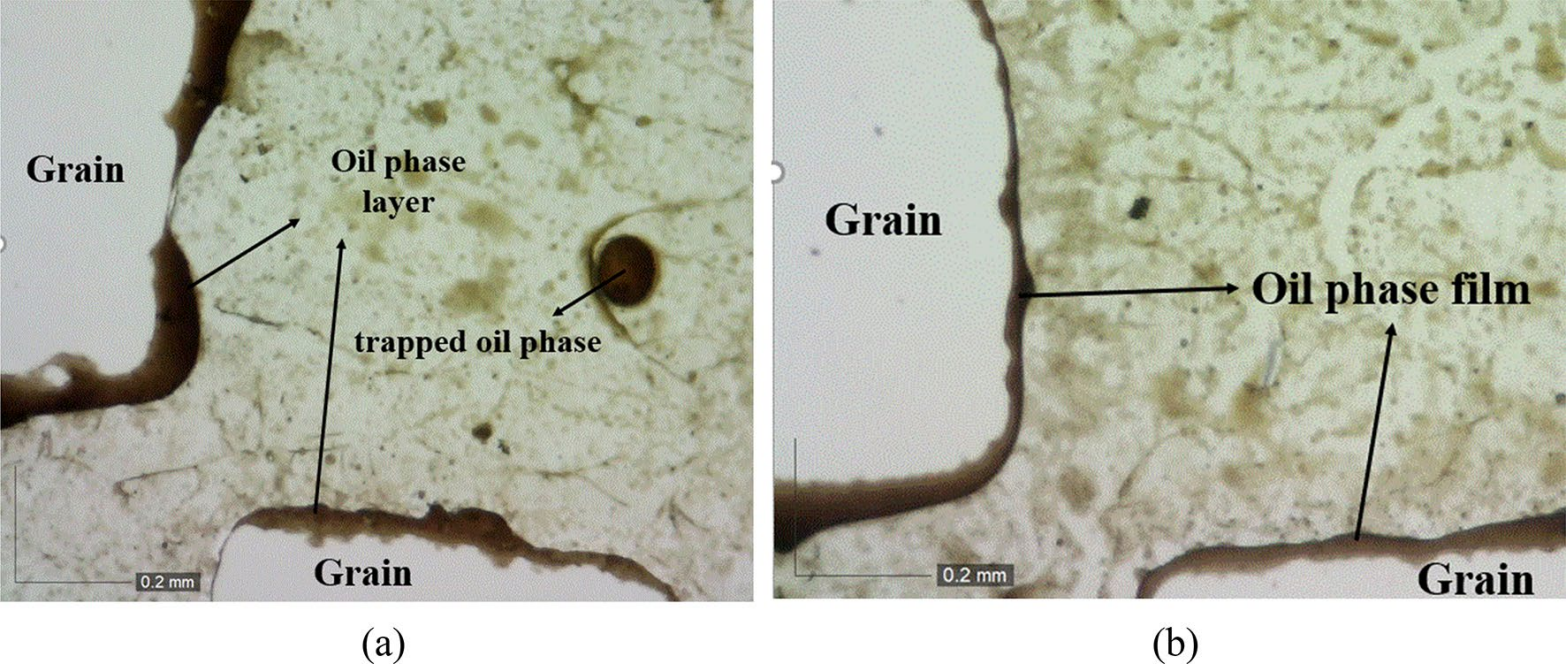
Microscopic images illustrating fluid flow paths in the first scenario during the initial (**a**) and subsequent (**b**) stages of injection.

In this section, high-resolution microscopic images are employed for a more detailed investigation and deeper understanding to explore the impact of the injection sequence on alterations in displacement of oil. Figure 9a displays a segment of the micromodel where a significant amount of oil has been carried away by smart water (Sw2K4SO_4_). Smart water, composed of a tailored formulation with specific ions like sulfate, exhibits enhanced oil recovery properties due to its ability to alter the wettability of the porous medium and improve oil displacement. Following the steps outlined for the first scenario, seawater injection into the porous medium is executed upon reaching the breakthrough stage. Seawater injection, although a common practice in oil recovery operations due to its availability and cost-effectiveness, does not manifest a discernible effect on the augmentation of oil recovery, as depicted in Fig. 9b.

**Figure 10:**
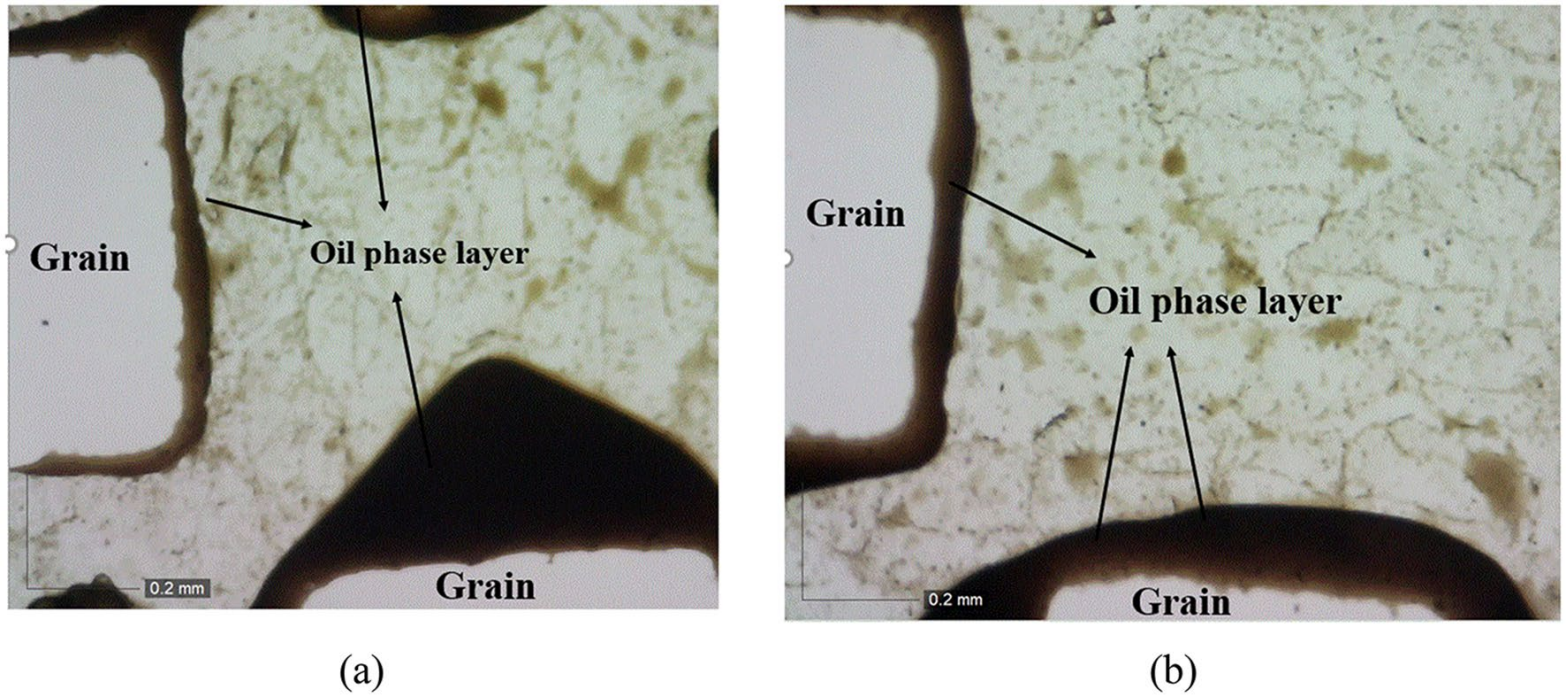
Microscopic images illustrating fluid flow paths in the second scenario during the initial (**a**) and subsequent (**b**) stages of injection.

Subsequently, the microscopic changes of phases during the initial injection of seawater into the porous medium will be examined. In Fig. 10a, when the second injection scenario into the micromodel is implemented, a significant portion of the oil remains in the porous medium during the seawater injection stage. This retention of oil can be attributed to factors such as interfacial tension between oil and water phases, capillary forces, and wettability characteristics of the rock surface. Meanwhile, as the second stage of injection with smart water (using the previous formulation) progresses, a notable amount of oil is separated and recovered from inside the micromodel. This observation aligns with the results presented in Fig. 10a, b, confirming the efficacy of smart water in displacing and recovering oil from porous media. The dynamic interaction between injected fluids and reservoir rock at the microscopic level elucidates the mechanisms governing enhanced oil recovery processes and underscores the importance of tailored fluid formulations in optimizing production efficiency.

In light of the compelling micromodel experiments, the observed trend of higher final recovery rates with smart water flooding followed by seawater flooding underscores the potential practical implications for field applications. While the subsequent seawater flooding yields a relatively modest increase in recovery, the substantial improvement achieved during the initial phase is noteworthy. This suggests that the sequential injection strategy, particularly with smart water as the primary agent, can be strategically applied in field scenarios to enhance oil extraction efficiency. The observed dominance of sulfate ions over potassium ions in altering wettability, as well as the nuanced relationship between sulfate and potassium ion concentrations, provides valuable insights for tailoring injection fluid compositions in real-world applications. Further exploration under field-simulated conditions, such as core-flooding experiments, may offer additional validation and guide the optimization of injection strategies for enhanced oil recovery at a larger scale.

## Conclusions

This study systematically explored the sequential injection of smart water and seawater to enhance oil extraction, uncovering nuanced insights into the underlying mechanisms governing wettability alteration and recovery efficiency. The following results were obtained:

- The unequivocal dominance of sulfate ions over potassium ions in inducing wettability alterations was established. The concentration gradient of sulfate ions exhibited a direct and proportional relationship with improved wettability towards hydrophilicity, underscoring sulfate ions as pivotal agents in surface interaction phenomena.
- The investigation revealed distinct mechanisms governing contact angle variation at varying sulfate and potassium ion concentrations. At sulfate ion concentrations of 0, 1, and 2 times, the optimal contact angle was achieved at 4 times the potassium ion concentration. This finding elucidates the intricate interplay of ionic strength and charge interactions on the mineral surface. Conversely, at concentrations of 3, 4, and 5 times sulfate ions, the optimal contact angle occurred at 2 times potassium ion concentration, suggesting a mechanism intricately linked to the increasing ionicity of sulfate ions.
- In the first injection scenario, where smart water preceded seawater, an initial recovery of notable efficacy was achieved. However, the subsequent seawater injection stage yielded a marginal increase in recovery, remaining below 1.5%.
- In the second injection scenario, commencing with seawater followed by smart water, initial recovery levels ranged between 17 and 18.7%. Subsequently, a marked augmentation of 1.1–7.9% in the second stage underscored the strategic role of sulfate ions in enhancing recovery efficiency.

In conclusion, this comprehensive investigation not only refines our comprehension of sulfate-ion-mediated wettability alterations but also elevates the discourse on enhanced oil recovery strategies, paving the way for impactful and scientifically rigorous contributions to the field.

## Data Availability

The data will be provided upon request to the corresponding author of this article, A. H. Saeedi Dehaghani via email at asaeedi@modares.ac.ir.
